# PPARγ/NF‐κB and TGF‐β1/Smad pathway are involved in the anti‐fibrotic effects of levo‐tetrahydropalmatine on liver fibrosis

**DOI:** 10.1111/jcmm.16267

**Published:** 2021-01-12

**Authors:** Qiang Yu, Ping Cheng, Jianye Wu, Chuanyong Guo

**Affiliations:** ^1^ Department of Gastroenterology Putuo People's Hospital Tongji University School of Medicine Shanghai China; ^2^ Department of Gastroenterology Shanghai Tenth People’s Hospital Tongji University School of Medicine Shanghai China; ^3^ Department of Gerontology Shanghai Minhang District Central Hospital Shanghai China

**Keywords:** autophagy, levo‐tetrahydropalmatine, liver fibrosis, PPARγ/NF‐κB, TGF‐β1/Smad

## Abstract

Liver fibrosis is a necessary stage in the development of chronic liver diseases to liver cirrhosis. This study aims to investigate the anti‐fibrotic effects of levo‐tetrahydropalmatine (L‐THP) on hepatic fibrosis in mice and cell models and its underlying mechanisms. Two mouse hepatic fibrosis models were generated in male C57 mice by intraperitoneal injection of carbon tetrachloride (CCl4) for 2 months and bile duct ligation (BDL) for 14 days. Levo‐tetrahydropalmatine was administered orally at doses of 20 and 40 mg/kg. An activated LX2 cell model induced by TGF‐β1 was also generated. The results showed that levo‐tetrahydropalmatine alleviated liver fibrosis by inhibiting the formation of extracellular matrix (ECM) and regulating the balance between TIMP1 and MMP2 in the two mice liver fibrosis models and cell model. Levo‐tetrahydropalmatine inhibited activation and autophagy of hepatic stellate cells (HSCs) by modulating PPARγ/NF‐κB and TGF‐β1/Smad pathway in vivo and in vitro. In conclusion, levo‐tetrahydropalmatine attenuated liver fibrosis by inhibiting ECM deposition and HSCs autophagy via modulation of PPARγ/NF‐κB and TGF‐β1/Smad pathway.

## 1 INTRODUCTION

Liver fibrosis is a wound‐healing response that can be induced by a variety of pathological factors, including viral infection, drug abuse, alcohol abuse, metabolic diseases, cholestatic diseases and autoimmune diseases.^1^, ^2^ Liver fibrosis is considered a necessary stage in the development of liver cirrhosis which may develop into hepatocellular carcinoma. In this progression, liver fibrosis is the reversible stage.^3^ In recent years, the worldwide morbidity and mortality of liver fibrosis is increasing, which has become a global health burden.^4^ The current treatment strategy for liver fibrosis includes antiviral agents, antioxidant agents, immunosuppressive agents and liver transplantation, which are limited by the side effects, high costs and lack of donors.^5^, ^6^ Hence, effective and safe anti‐fibrotic agents are urgently needed.

Liver fibrosis is characterized by the excessive deposition of extracellular matrix (ECM) in the subendothelial space.^7^ Matrix metalloproteinases (MMPs) regulate the degradation of ECM, tissue inhibitors of metalloproteinases (TIMPS) promote synthesis and inhibit the degradation of ECM. In physiological conditions, the dynamic balance between MMPs and TIMPs maintains the ECM homoeostasis in the liver, which is disturbed in liver fibrosis.^8^

Hepatic stellate cells (HSCs) are the main source of ECM in the liver. The activation of HSCs plays a key role in the pathogenesis of liver fibrosis and is considered the initial stage.^9^ In normal conditions, HSCs store retinoid and vitamin A in the liver.^10^ During liver injury, HSCs are activated by autocrine or paracrine stimuli and transform into proliferative and contractile myofibroblasts (MFBs), along with excessive production of collagen type I (Col‐1), the main ingredient of ECM. Activated HSCs also express alpha‐smooth muscle actin (α‐SMA) and platelet‐derived growth factor receptor (PDGFR).^11^ The release of transforming growth factor (TGF)‐β1 and other inflammatory factors by Kupffer cells, sinusoidal endothelial cells and other inflammatory cells goes along with the activation during liver injury.^12^ TGF‐β1 pathway plays a significant role in the pathogenesis of liver fibrosis. On the one hand, elevated expression of TGF‐β1 results in the continuous activation of HSCs. On the other hand, TGF‐β1modulates the expression of MMPs and TIMPs.^13^ It has been reported that inhibition of TGF‐β1 could suppress the activation of HSCs in vivo and in vitro.^14^

Autophagy, or type II programmed cell death refers to the phenomenon that cells digest non‐functioning and damaged organelles, protein aggregates and invading microbes to provide energy and promote cell survival during hypoxia, infection, and starvation.^15^ The roles of autophagy in autoimmune hepatitis, liver ischaemia‐reperfusion injury, liver fibrosis and hepatocellular carcinoma have been demonstrated.^16^, ^17^, ^18^, ^19^ It has been reported that HSC autophagy could provide energy for HSC activation by degrading lipids.^20^ The suppression of HSC autophagy ameliorates HSC activation, production of ECM and fibrogenesis in vivo and in vitro.^20^, ^21^ Therefore, autophagy is considered a therapeutic target for liver fibrosis.

Levo‐tetrahydropalmatine (L‐THP) is an active component excreted from traditional Chinese medicine, *Corydalis yanhusuo*. L‐THP has been reported to have a sedative effect, analgesic effect, cardioprotective effect, antioxidative effect and antitumor effect.^22^, ^23^, ^24^, ^25^, ^26^ Our previous studies demonstrated that L‐THP could suppress apoptosis and autophagy in the presence of liver injury induced by concanavalin A (ConA) and hepatic ischaemia and reperfusion (IR).^27^, ^28^ ConA injection and hepatic IR can both result in the activation of HSCs.^29^, ^30^ Moreover, dl‐tetrahydropalmatine (dl‐THP), another active component of *Corydalis yanhusuo*, was reported to ameliorate CCl4‐induced liver injury in mice by inhibiting hepatic lipoperoxidation.^31^

Based on the findings above, we hypothesized that L‐THP could suppress liver fibrosis by inhibiting HSCs autophagy. In the present study, mice liver fibrosis models induced by CCl4 and bile duct ligation (BDL) and an activated LX2 cell model were generated to verify the anti‐fibrotic effect of L‐THP. The underlying mechanisms were also investigated, focusing on PPARγ/NF‐κB and TGF‐β1/Smad pathway.

## 2 MATERIALS AND METHODS

### 2.1 Reagents

Levo‐tetrahydropalmatine (L‐THP), dimethyl sulphoxide (DMSO), sodium pentobarbital and carbon tetrachloride (CCl4) were purchased from Sigma‐Aldrich Co. (St Louis, MO, USA). The kits for detecting alanine aminotransferase (ALT), hydroxyproline, and aspartate aminotransferase (AST) were purchased from Jiancheng Bioengineering Institute (Nanjing, China). Reverse transcription kit, polymerase chain reaction (PCR) kit and Cell Counting Kit 8 (CCK‐8) were bought from Epizyme Biotech (Shanghai, China). The primers were purchased from Generay (Shanghai, China). The primary antibodies against TIMP1 (1:1000), MMP2 (1:1000), Beclin1 (1:1000), LC3 (1:1000), p62 (1:1000), IκBα (1:1000), Smad2 (1:1000) were purchased from Proteintech (Chicago, IL, USA). Antibodies against Col‐1 (1:1000), p‐Smad2 (1:1000) and p‐Smad3 (1:2000) were obtained from Abcam (Cambridge, MA, USA). Antibodies against β‐actin (1:1000), NF‐κB (1:1000) and Smad3 (1:1000) were purchased from Cell Signaling Technology (Danvers, MA, USA). Antibody against α‐SMA (1:100) was purchased from Servicebio (Wuhan, China). Antibody against PPARγ (1:1000) was purchased from ABclonal (Wuhan, China). Antibody against TGF‐β1 (1:1000) was obtained from Bioworld Technology (Shanghai, China). High glucose Dulbecco’s Modified Eagle Medium(DMEM) and foetal bovine serum (FBS) were purchased from HyClone (GE Healthcare, Logan, UT, USA). Human TGF‐β1 was purchased from PeproTech (Suzhou, China).

### 2.2 Animals

Healthy 6‐8‐week‐old male C57 mice (23 ± 2 g) were purchased from Shanghai Laboratory Animal. Co., Ltd. (Shanghai, China). The mice were raised in tidy cages in the animal house with a 24°C ± 2°C temperature and a 12 hours light/dark cycle. The mice had free access to clean drinking water and mice feed. All the animal experiments in the present were carried out following the National Institutes of Health Guidelines and approved by the Animal Care and Use Committee of Shanghai Tongji University (Shanghai, China).

### 2.3 Establishment of two different liver fibrosis mouse models

Carbon tetrachloride (CCl4) was diluted with olive oil to 10%. L‐THP was diluted with 1% DMSO to 20 and 40 mg/mL, this solution was administrated by gavage. Sixty male C57 mice were divided randomly into ten groups (six mice per group) as follows:

1. Normal control (NC) (n = 6), mice underwent no treatment;
2. Vehicle (n = 6), mice were injected intraperitoneally with 1 ml/kg olive oil three times a week for 8 weeks;
3. L‐THP (40) (n = 6), mice were administrated with 40 mg/kg L‐THP by gavage three times a week for 8 weeks;
4. CCl4 (n = 6), mice were injected intraperitoneally with 1 mL/kg 10% CCl4 three times a week for 8 weeks;
5. CCl4+L‐THP (20) (n = 6), mice were injected intraperitoneally with 1 mL/kg 10% CCl4 and administrated with 20 mg/kg L‐THP by gavage three times a week for 8 weeks;
6. CCl4+L‐THP (40) (n = 6), mice were injected intraperitoneally with 1 mL/kg 10% CCl4 and administrated with 40 mg/kg L‐THP by gavage three times a week for 8 weeks;
7. Sham (n = 6), mice underwent laparotomy without bile duct ligation and were maintained for 14 days;
8. BDL (n = 6), mice underwent bile duct ligation (BDL) surgery and were maintained for 14 days;
9. BDL+L‐THP (20) (n = 6), mice were administrated with 20 mg/kg L‐THP by gavage once daily for 14 days after BDL surgery;
10. BDL+L‐THP (40) (n = 6), mice were administrated with 40 mg/kg L‐THP by gavage once daily for 14 days after BDL surgery.

In group 8, 9 and 10, mice underwent bile duct ligation (BDL) surgery as previously described.^1^ In brief, the mice were anaesthetized with 1.25% sodium pentobarbital by intraperitoneal injection. Then, the abdomen was opened via linea alba. The bile duct was exposed and isolated from the flank portal vein and hepatic artery. The bile duct was tied with two surgical knots and cut between the two knots. The abdomen was closed and the mice were placed on a warm blanket before awakening.

At the end of the model establishment, the mice were sacrificed by CO_2_ inhalation. The livers and blood samples were collected. Blood samples underwent centrifugation (4°C, 4300 *g*, 10 minutes) to acquire serum. The serum samples and livers were stored at −80°C.

### 2.4 Biochemical analysis

Serum levels of aminotransferase (ALT) and aspartate aminotransferase (AST) were detected by an automated chemical analyser (Olympus AU1000; Olympus, Tokyo, Japan). Hepatic levels of hydroxyproline were determined using a commercial kit following the manufacturer’s instructions.

### 2.5 Histological analysis

Fresh liver lobes were washed with normal saline and then fixed with 4% paraformaldehyde for 24 hours at 4°C. Then, the livers were embedded in paraffin and cut into 5 μm‐thick sections. The sections were then stained with haematoxylin and eosin (H&E) to determine liver injury, and with Masson’s trichrome (MT) to determine collagen deposition.

### 2.6 Real‐time polymerase chain reaction

Total RNA was acquired from the frozen liver tissues or LX2 cells using a TRIzol reagent (Tiangen Biotech, Beijing, China). Total RNA was reverse transcribed into cDNA using a reverse transcription kit (Epizyme Biotech, Shanghai, China). Then, real‐time polymerase chain reaction (RT‐PCR) was performed using a Hieff qPCR SYBR Green Master Mix (Epizyme Biotech, Shanghai, China). The target gene expression was detected by a 7900HT fast real‐time PCR system (ABI, Foster City, CA, USA). The primers used in the present study are listed in Table 1 (Mouse) and Table S1 (Human).

**TABLE 1:** Sequences of primers (Mouse) used for RT‐PCR

Gene	DNA strand	Primer sequence (5′‐3′)
β‐actin	Forward Reverse	GGCTGTATTCCCCTCCATCG CCAGTTGGTAACAATGCCATGT
Col‐1(α1)	Forward Reverse	CAATGGCACGGCTGTGTGCG AGCACTCGCCCTCCCGTCTT
α‐SMA	Forward Reverse	CCCAGACATCAGGGAGTAATGG TCTATCGGATACTTCAGCGTCA
TIMP1	Forward Reverse	CGAGACCACCTTATACCAGCG ATGACTGGGGTGTAGGCGTA
MMP2	Forward Reverse	GGACAAGTGGTCCGTGTAAA CCGACCGTTGAACAGGAAGG
Beclin‐1	Forward Reverse	ATGGAGGGGTCTAAGGCGTC TGGGCTGTGGTAAGTAATGGA
LC3	Forward Reverse	GACCGCTGTAAGGAGGTGC AGAAGCCGAAGGTTTCTTGGG
NF‐κB	Forward Reverse	ATGGCAGACGATGATCCCTAC CGGATCGAAATCCCCTCTGTT
PPARγ	Forward Reverse	GGAAGACCACTCGCATTCCTT GTAATCAGCAACCATTGGGTCA
TGF‐β1	Forward Reverse	CCACCTGCAAGACCATCGAC CTGGCGAGCCTTAGTTTGGAC

### 2.7 Western blotting analysis

Total protein was extracted from frozen liver tissues or LX2 cells using radioimmunoprecipitation assay lysis (PIPA) buffer added with protease inhibitors (PI) and phenylmethanesulfonyl fluoride (PMSF). The protein concentration was determined with a bicinchoninic acid assay (BCA). Then, equal quality of protein samples was electrophoresed on 7.5%‐12.5% sodium dodecyl sulphate polyacrylamide gels (SDS‐PAGE). The protein samples were then transferred onto polyvinylidene fluoride (PVDF) membranes or nitrocellulose (NC) membranes. Then, the blots were blocked by 5% defatted milk powder or 5% bovine serum albumin (BSA) for 1 hour at room temperature. Then, the members were incubated with specific primary antibodies and secondary bodies. An Odyssey two‐colour infrared laser imaging system (LI‐COR Biosciences) was employed to scan the blots. The quantitative analysis of blot’s grey value was performed using ImageJ software.

### 2.8 Immunohistochemical staining

Paraffin‐embedded 5‐μm liver sections were dewaxed and dehydrated with different concentrations of ethanol. After an antigen retrieval process, the sections were added with 3% hydrogen peroxide (H_2_O_2_) solution to block endogenous peroxidase activity. Then, the sections were incubated in 5% bovine serum albumin (BSA) to block non‐specific proteins. Antibodies, including anti‐Col‐1; anti‐α‐SMA; anti‐Beclin1; anti‐LC3; anti‐PPARγ; anti‐NF‐κB; anti‐TGF‐β1; anti‐p‐Smad2; anti‐p‐Smad3 were added to the sections and then incubated for 12 hours at 4°C. The sections were then incubated with specific secondary antibodies and a peroxidase substrate (DAB) kit. The positive areas were observed using light microscopy.

### 2.9 Transmission electron microscopy

Liver sections were cut into 1 mm^3^ fragment and then fixed in 2% glutaraldehyde buffered with 0.2 mmol/L cacodylates. Then, the sections were post‐fixed with osmium tetroxide and embedded in epoxy resin. Autophagosomes were observed using electron microscopy (JEM‐1230; JEOL, Tokyo, Japan).

### 2.10 Double‐immunofluorescence staining

Paraffin‐embedded 5‐μm liver sections were dewaxed and dehydrated. After an antigen retrieval process, the sections were incubated with 3% bovine serum albumin (BSA) for 30 minutes. Then, the liver sections were incubated with α‐SMA (green) and Beclin1 (red) or LC3 (red) primary antibodies (1:500) at 4°C overnight. The liver sections were then incubated with fluorescence secondary antibody, the nucleus was stained with DAPI. After mounted with antifluorescence quenching sealant, the liver sections were observed using inverted fluorescence microscope (DMIRB; Leica Microsystems, Wetzlar, Germany).

### 2.11 Cell culture and CCK8 assay

Human immortal HSC cell line LX‐2 was bought from Cell Bank of Type Culture Collection of the Chinese Academy of Sciences. LX‐2 cells were cultured in high glucose Dulbecco’s modified Eagle’s medium (DMEM) with 10% foetal bovine serum (FBS), 100 U/mL penicillin and 100 g/mL streptomycin. To be activated, LX‐2 cells were added with exogenous 10 ng/mL TGF‐β1 and incubated for at least 24 hours as previously described.^14^ To detect the effect of L‐THP on the proliferation of quiescent and activated LX‐2 cells in vitro, logarithmic phase LX‐2 cells were seeded in 96‐well plates with or without 10 ng/mL TGF‐β1. After 48 hours, the cells were added with 0, 20, 40, 80, 160, 320 μmol/L L‐THP and incubated for 24 hours. Then, a CCK8 assay was performed to detect the cell viability. The half‐maximum inhibition concentration (IC50) of L‐THP was calculated with CalsuSyn software.

### 2.12 Detection of effects of L‐THP on activated HSCs in vitro

To further detect the effects of L‐THP on the ECM synthesis, autophagy and TGF‐β1/Smad and PPARγ/NF‐κB pathway in activated HSCs in vitro, LX‐2 cells seeded in 6‐well plates were divided into the following three groups: (a) Control group: LX‐2 with no additional treatments; (b) TGF‐β1 group: LX‐2 cells were treated with 10 ng/mL TGF‐β1; (c) L‐THP group: LX‐2 cells were treated with 10 ng/mL TGF‐β1 and L‐THP at a dose of IC50. After 72 hours, the total proteins of LX‐2 cells were collected and the protein expressions of Col‐1, α‐SMA, TIMP1, MMP2, Beclin1, LC3 and P62 were detected by Western blotting.

### 2.13 Statistical analysis

All experiments were repeated at least three times. The results were presented as means ± standard deviation (SD). The student *t‐*test was performed to conduct statistical analysis and *P*‐values < .05 were considered statistically significant.

## 3 RESULTS

### 3.1 L‐THP, olive oil and laparotomy had no side effects on normal liver function

**FIGURE 1:**
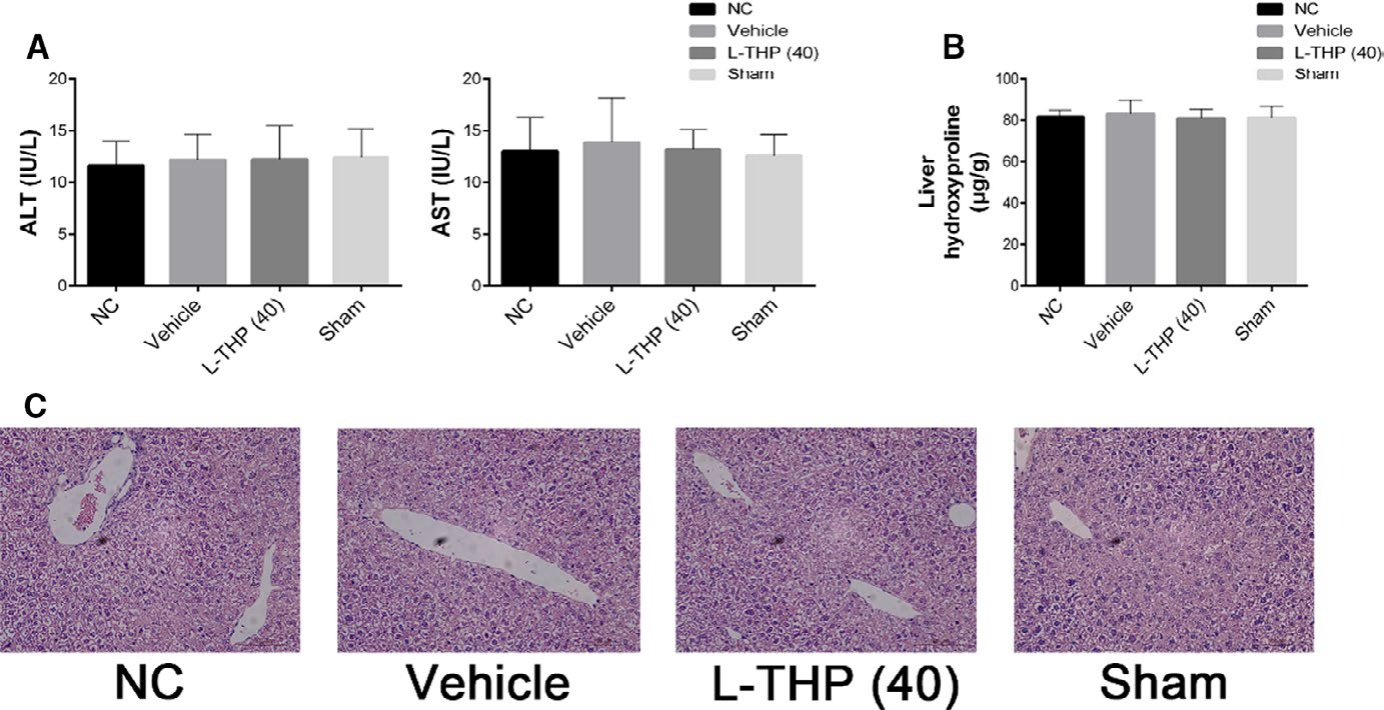
Effects of olive oil, L‐THP (40 mg/kg), and laparotomy on normal liver tissues. A, The levels of serum ALT and AST in the four groups did not differ. Data were given as means ± SD (n = 6, *P* > .05). B, Hepatic levels of hydroxyproline in the four groups did not differ; data are given as means ± SD (n = 6, *P* > .05). C, H&E stains of liver sections in the four groups (Original magnification: ×400)

The serum levels of liver enzymes (ALT and AST) reflect the severity of liver injury. Hydroxyproline is a marker of collagen synthesis and can indicate the extent of liver fibrosis. No significant difference was detected in the serum levels of ALT, AST and hepatic levels of hydroxyproline between the NC group, L‐THP (40) group, Vehicle group and Sham group (Figure 1A & B). Moreover, no obvious histological changes were observed in the H&E‐stained liver sections from these four groups (Figure 1C). These findings demonstrated that L‐THP (40 mg/kg), olive oil and laparotomy did not affect normal mice liver functions. Vehicle group and Sham group were employed as the control group in the two liver fibrosis models respectively in the next experiments.

### 3.2 L‐THP ameliorated liver fibrosis in mice models induced by CCl4 and BDL

**FIGURE 2:**
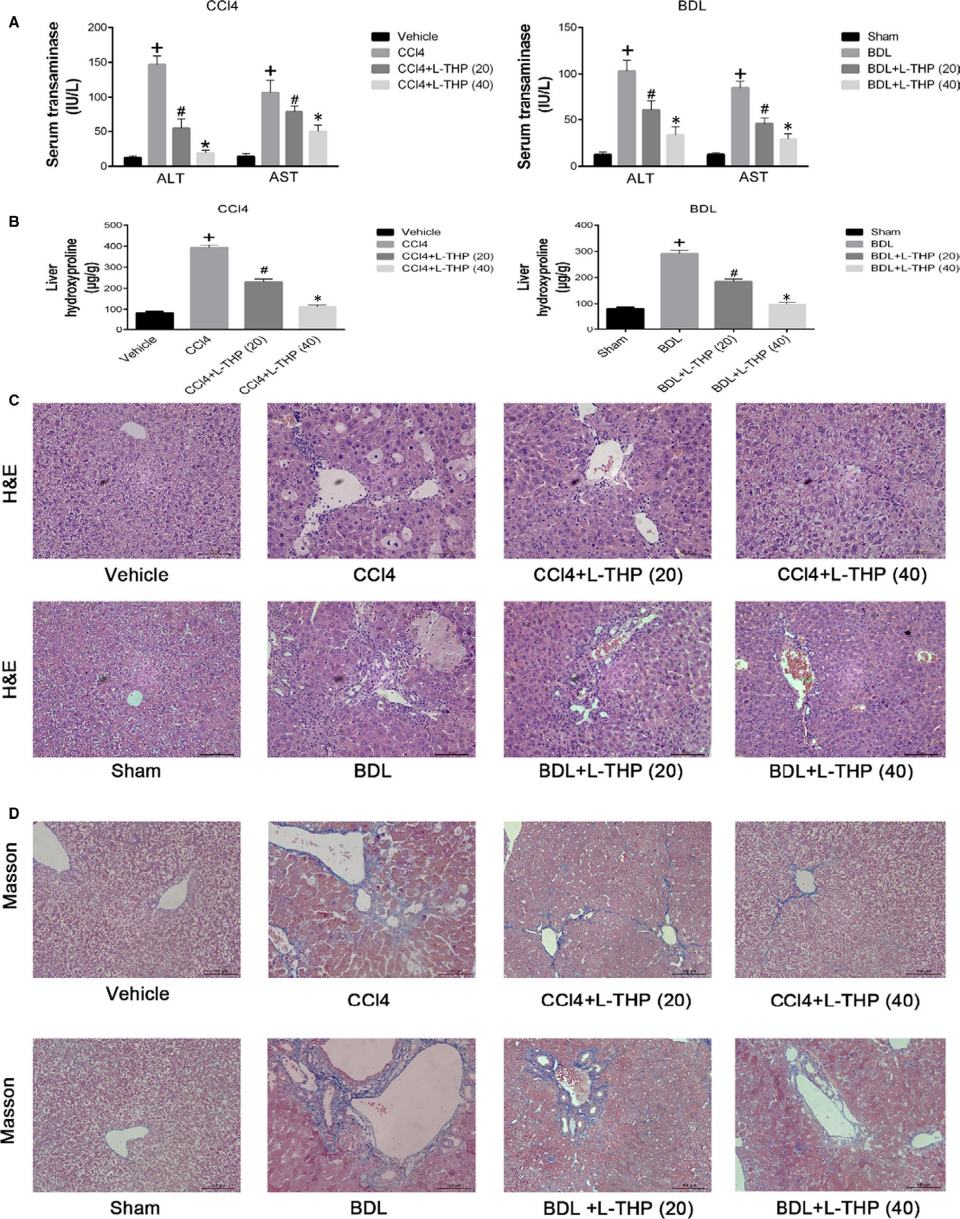
Effects of levo‐tetrahydropalmatine on liver function and pathological changes induced by CCl4 and BDL: (A and B) L‐THP decreased the serum levels of ALT, AST and hepatic levels of hydroxyproline induced by CCl4 and BDL in a dose‐dependent manner. Data are expressed as means ± SD (n = 6,^+^ *P* < .05 for CCl4 or BDL versus Vehicle or Sham; *^#^P* for CCl4+L‐THP (20) or BDL+L‐THP (20) versus CCl4 or BDL; *P for CCl4 +L‐THP (40) or BDL+L‐THP (40) versus CCl4 +L‐THP (20) or BDL+L‐THP (20)). (C and D) L‐THP ameliorated hepatic pathological change, showed by H&E and Masson’s staining (original magnification: ×400)

Compared with Vehicle and Sham groups, the serum transaminase levels in CCl4 and BDL groups increased significantly. L‐THP treatment (20/40 mg/kg) reduced the serum transaminase levels, 40 mg/kg L‐THP had a greater protective effect (Figure 2A).

The hepatic hydroxyproline levels increased significantly in CCl4 and BDL groups compared with Vehicle and Sham groups, which were reduced by L‐THP treatment dose‐dependently (Figure 2B). The histological changes in the livers were evaluated by H&E and Masson staining. As shown in Figure 2C, CCl4 administration and BDL resulted in hepatocyte ballooning and necrosis, inflammatory infiltration, and reconstruction of the hepatic lobule structure and bile duct proliferation. The histological changes in both liver fibrosis models were alleviated by the L‐THP treatment. The 40 mg/kg L‐THP showed a greater protective effect. Masson staining indicated an increase of blue areas with collagen fibres in liver sections from the CCl4 and BDL groups, which was ameliorated by L‐THP treatment in dose‐dependent manners (Figure 2D).

### 3.3 L‐THP inhibited the formation of ECM and HSCs activation

**FIGURE 3:**
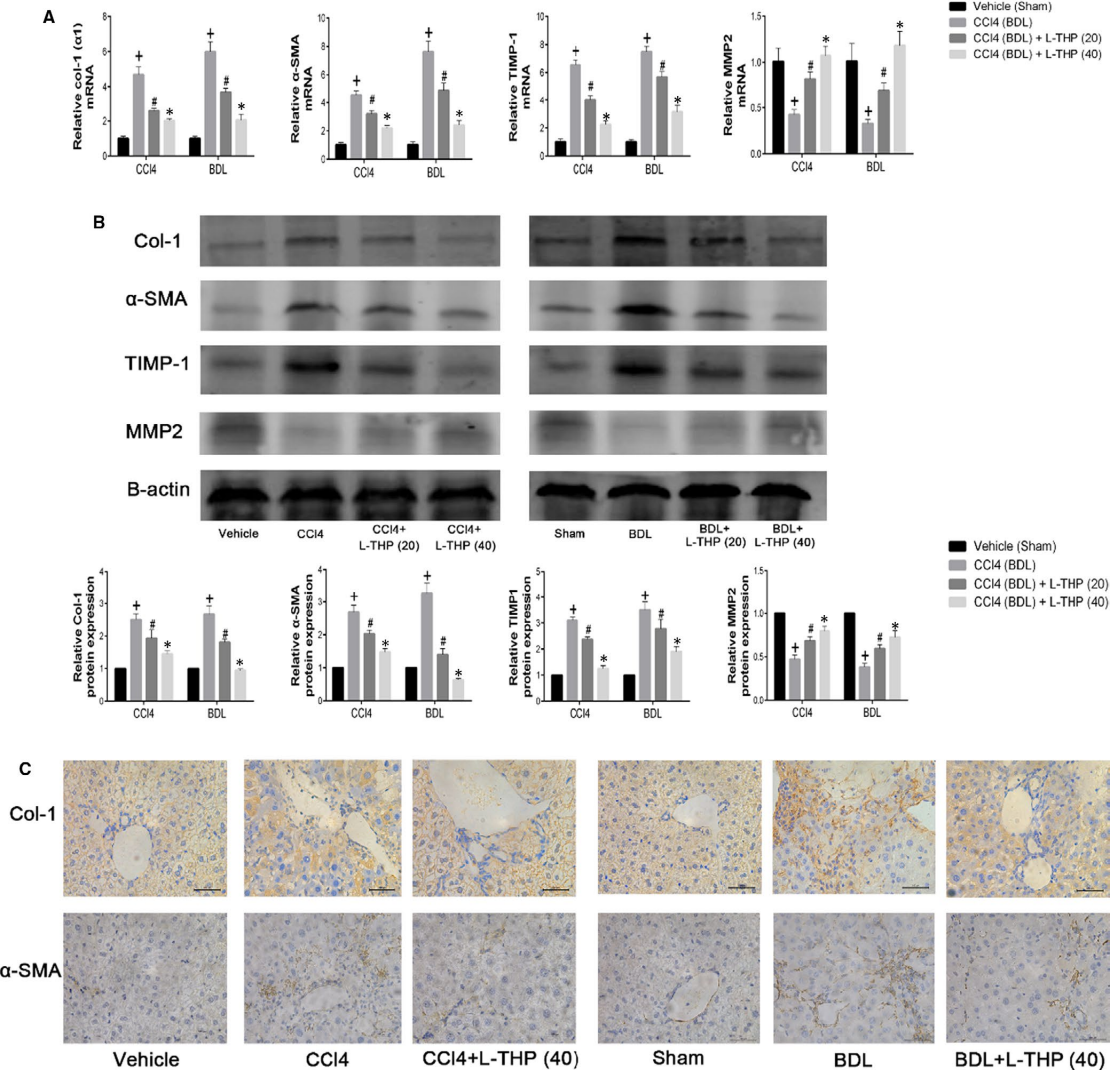
Effects of levo‐tetrahydropalmatine on ECM and HSCs activation in liver fibrosis. A, The PCR analysis. Levo‐tetrahydropalmatine reduced mRNA expressions of Col‐1 (α1), α‐SMA, and TIMP1 and increased mRNA expressions of MMP2 in the two mice liver fibrosis models. Data are expressed as means ± SD (n = 3,^+^ *P* < .05 for CCl4 or BDL versus Vehicle or Sham; *^#^P* for CCl4+L‐THP (20) or BDL+L‐THP (20) versus CCl4 or BDL; *P for CCl4 +L‐THP (40) or BDL+L‐THP (40) versus CCl4 +L‐THP (20) or BDL+L‐THP (20)). B, Western blot and quantitative analysis. Levo‐tetrahydropalmatine treatment significantly reduced the protein expressions of Col‐1, α‐SMA, and TIMP1 and increased the protein expression of MMP2. Data are expressed as means ± SD (n = 3,^+^ *P* < .05 for CCl4 or BDL versus Vehicle or Sham; *^#^P* for CCl4+L‐THP (20) or BDL+L‐THP (20) vs CCl4 or BDL; *P for CCl4 +L‐THP (40) or BDL+L‐THP (40) vs CCl4 +L‐THP (20) or BDL+L‐THP (20)). C, Immunohistochemical staining indicated that the increased protein expressions of Col‐1, α‐SMA in CCl4 and BDL groups were suppressed by 40 mg/kg L‐THP treatment (original magnification: ×400)

Liver fibrosis is characterized by the activation of HSCs and the over deposition of extracellular matrix (ECM). Collagen I (Col‐1) is the main composition of ECM, whose synthesis and degradation are modulated by matrix metalloproteinases (MMPs) and tissue inhibitors of matrix metalloproteinase (TIMPs). α‐SMA is considered a marker of HSCs activation and the expression of ECM. In the present study, the effects of L‐THP treatment on HSCs activation and ECM production in liver fibrosis were assessed by determining mRNA and protein expressions of Col‐1, α‐SMA, TIMP1 and MMP2 in the liver tissues from the two different liver fibrosis models. The results of PCR indicated that Col‐1 (α1), α‐SMA and TIMP1 mRNA levels in CCl4 and BDL groups were significantly higher than that in Vehicle and Sham groups, and this trend was inhibited by L‐THP treatment in dose‐dependent manners. The MMP2 mRNA levels in CCl4 and BDL groups were significantly lower than that in Vehicle and Sham groups, which was reversed by L‐THP treatment (Figure 3A). Moreover, the results of western blotting demonstrated that the protein expressions of Col‐1, α‐SMA and TIMP1 were enhanced by CCl4 and BDL, which were suppressed by L‐THP treatment dose‐dependently (Figure 3B). The protein expressions of MMP2 were inhibited by CCl4 and BDL, while were reversed by L‐THP. And the 40 mg/kg L‐THP exerted a greater effect than 20 mg/kg. The protein expressions of Col‐1 and α‐SMA were further detected by immunohistochemical staining (Figure 3C). The results of immunohistochemical staining were in accordance with PCR and western blotting. These results demonstrated that L‐THP suppressed the HSCs activation and ECM formation to ameliorate liver fibrosis.

### 3.4 L‐THP inhibited autophagy in both liver fibrosis models

**FIGURE 4:**
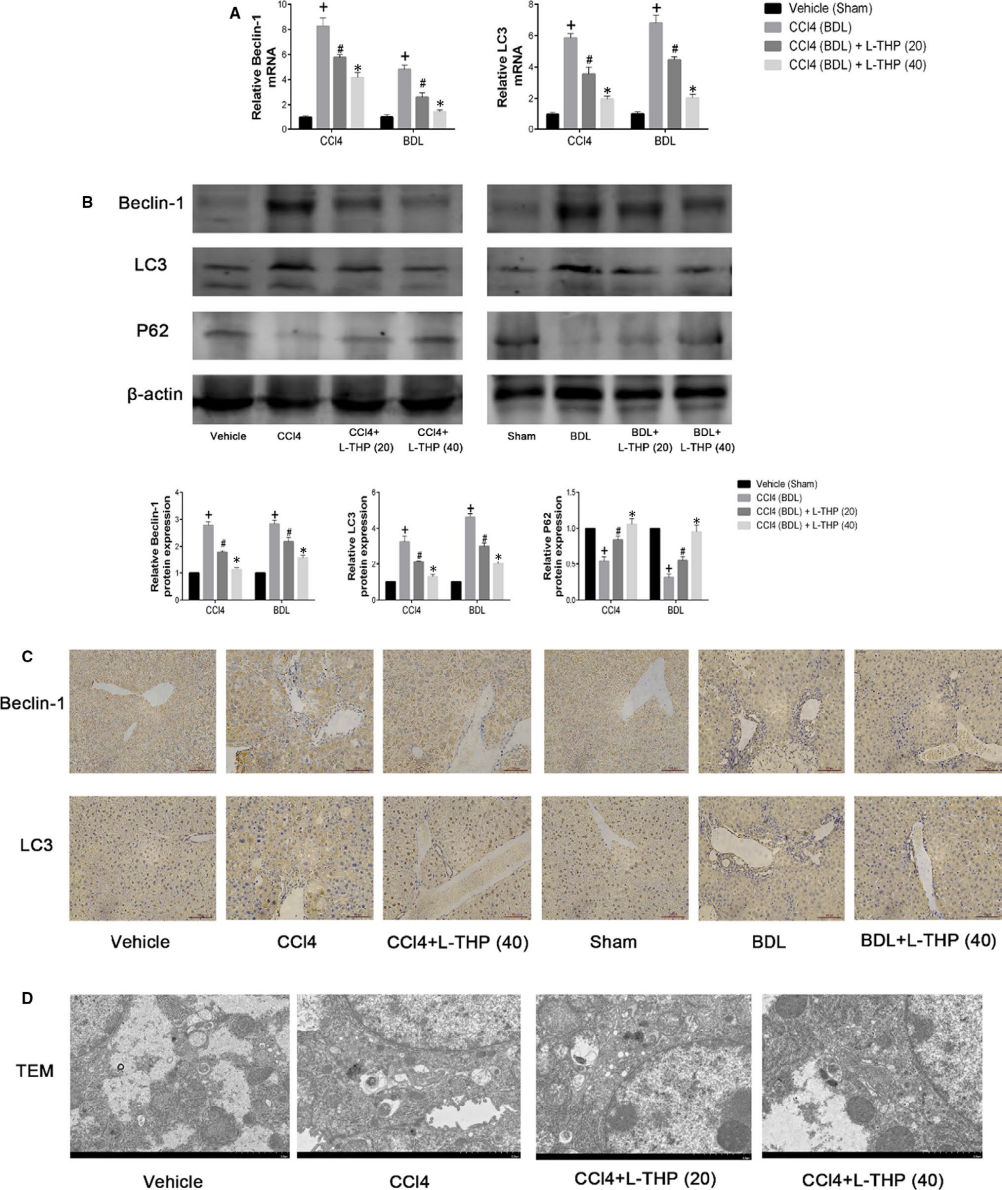
Effects of levo‐tetrahydropalmatine on autophagy in liver fibrosis. A, The PCR analysis. The mRNA levels of Beclin1 and LC3 were significantly down‐regulated by L‐THP. Data are expressed as means ± SD (n = 3, ^+^ *P* < .05 for CCl4 or BDL vs Vehicle or Sham; *^#^P* for CCl4+L‐THP (20) or BDL+L‐THP (20) vs CCl4 or BDL; *P for CCl4 +L‐THP (40) or BDL+L‐THP (40) versus CCl4 +L‐THP (20) or BDL+L‐THP (20)). B, Western blot and quantitative analysis. L‐THP treatment significantly reduced the protein expressions of Beclin1 and LC3 and increased the protein expression of P62. Data are expressed as means ± SD (n = 3,^+^ *P* < 0.05 for CCl4 or BDL versus Vehicle or Sham; *^#^P* for CCl4+L‐THP (20) or BDL+L‐THP (20) versus CCl4 or BDL; *P for CCl4 +L‐THP (40) or BDL+L‐THP (40) vs CCl4 +L‐THP (20) or BDL+L‐THP (20)). C, The areas of positive cells of Beclin1 and LC3 were diminished by L‐THP as shown by immunohistochemistry staining (original magnification: ×400). D, L‐THP inhibited the autophagosome formation in the liver sections from the CCl4‐induced fibrosis model (original magnification: ×7000)

Autophagy could accelerate liver fibrosis by providing energy for HSCs activation. Our previous studies indicated that L‐THP inhibited autophagy to alleviate mice liver injury induced by ConA and hepatic ischaemia and reperfusion.^27^, ^28^ In the present study, the effects of L‐THP on autophagy levels in mice liver fibrosis were detected. Autophagy is characterized by the formation of autophagosomes.^20^ Beclin1 and LC3 promote the formation of autophagosomes. P62 is an autophagy‐related transporter, which is degraded during the formation of autophagosomes. Beclin1, LC3 and P62 are considered the biomarkers of cell autophagy. In the present study, the mRNA and protein levels of these autophagy‐related markers were detected by PCR, western blotting and immunohistochemical staining. The results of western blotting showed that compared to the Vehicle and BDL group, the protein expressions of Beclin1 and LC3 increased and P62 decreased in CCl4 and BDL groups, which were reversed by L‐THP treatment in dose‐dependent manners (Figure 4B). The results of PCR were consistent with western blotting (Figure 4A). Immunohistochemical staining indicated that the increased protein levels of Beclin1 and LC3 in the liver sections in CCl4 and BDL group were inhibited by 40 mg/kg L‐THP (Figure 4C). Furthermore, TEM was employed to observe the formation of autophagosomes in liver sections of the CCl4‐induced liver fibrosis model. Comparing with Vehicle group, more autophagosomes were observed in CCl4 group, which was suppressed by L‐THP treatment. 40 mg/kg L‐THP showed a greater suppressive effect on autophagosome formation than 20 mg/kg L‐THP (Figure 4D). In addition, to detect the effect of L‐THP treatment on the expression of autophagy‐related proteins in HSCs in the fibrotic liver specifically, double‐immunofluorescence staining was performed on the liver sections from CCl4‐treated groups. The results showed that 40 mg/kg L‐THP inhibited the protein expressions of Beclin1 and LC3 in HSCs (Figure S1). These results demonstrated that L‐THP suppressed HSCs autophagy in mice fibrotic livers.

### 3.5 Levo‐tetrahydropalmatine down‐regulated TGF‐β1/Smad pathway

**FIGURE 5:**
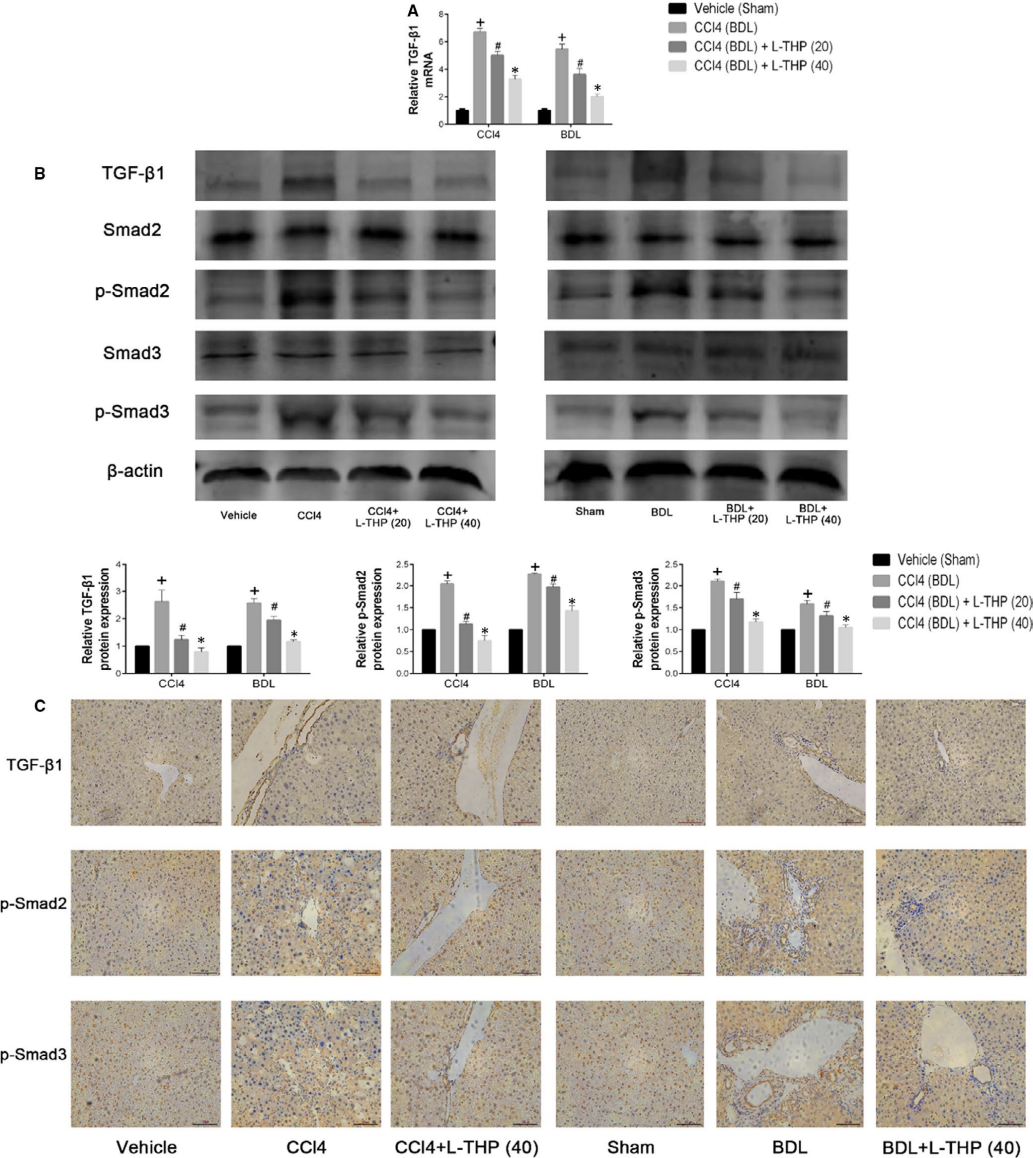
Effects of levo‐tetrahydropalmatine on the TGF‐β1/Smad pathway in liver fibrosis: (A) The PCR analysis. The mRNA levels of TGF‐β1were significantly down‐regulated by L‐THP. Data are expressed as means ± SD (n = 3,^+^ *P* < .05 for CCl4 or BDL vs Vehicle or Sham; *^#^P* for CCl4+L‐THP (20) or BDL+L‐THP (20) versus CCl4 or BDL; *P for CCl4 +L‐THP (40) or BDL+L‐THP (40) vs CCl4 +L‐THP (20) or BDL+L‐THP (20)). B, Western blot and quantitative analysis. L‐THP treatment significantly reduced the protein expressions of TGF‐β1, p‐Smad2 and p‐Smad3. Data are expressed as means ± SD (n = 3, ^+^ *P* < .05 for CCl4 or BDL vs Vehicle or Sham; *^#^P* for CCl4+L‐THP (20) or BDL+L‐THP (20) vs CCl4 or BDL; *P for CCl4 +L‐THP (40) or BDL+L‐THP (40) vs CCl4 +L‐THP (20) or BDL+L‐THP (20)). C, Immunohistochemical staining indicated that the increased protein expressions of TGF‐β1, p‐Smad2 and p‐Smad3 in CCl4 and BDL groups were suppressed by 40 mg/kg L‐THP treatment (original magnification: ×400)

TGF‐β1 is an inflammatory cytokine secreted by HSCs, Kupffer cells, hepatic sinus endothelial cells and inflammatory cells. TGF‐β1 could phosphorylate Smads and promote the translocation of phosphorylated Smads (p‐Smads) to the nuclear region. TGF‐β1/Smad pathway plays a significant role in the progression of liver fibrosis by modulating ECM synthesis and HSCs autophagy.^32^ The results of PCR showed that mRNA levels of TGF‐β1 were significantly higher in CCl4 and BDL groups comparing with Vehicle and Sham group, which were inhibited by L‐THP treatment dose‐dependently (Figure 5A). The results of western blot indicated that protein expressions of TGF‐β1, phosphorylated‐Smad2 (p‐Smad2) and phosphorylated‐Smad3 (p‐Smad3) were up‐regulated in Vehicle and BDL groups comparing with control groups, while were all suppressed by L‐THP treatment in dose‐dependent manners (Figure 5B). The results of immunohistochemistry were consistent with western blotting (Figure 5C). These results demonstrated that L‐THP treatment down‐regulated the TGF‐β1/Smad pathway in the two mice fibrosis models.

### 3.6 Levo‐tetrahydropalmatine up‐regulated PPARγ/NF‐κB signalling

**FIGURE 6:**
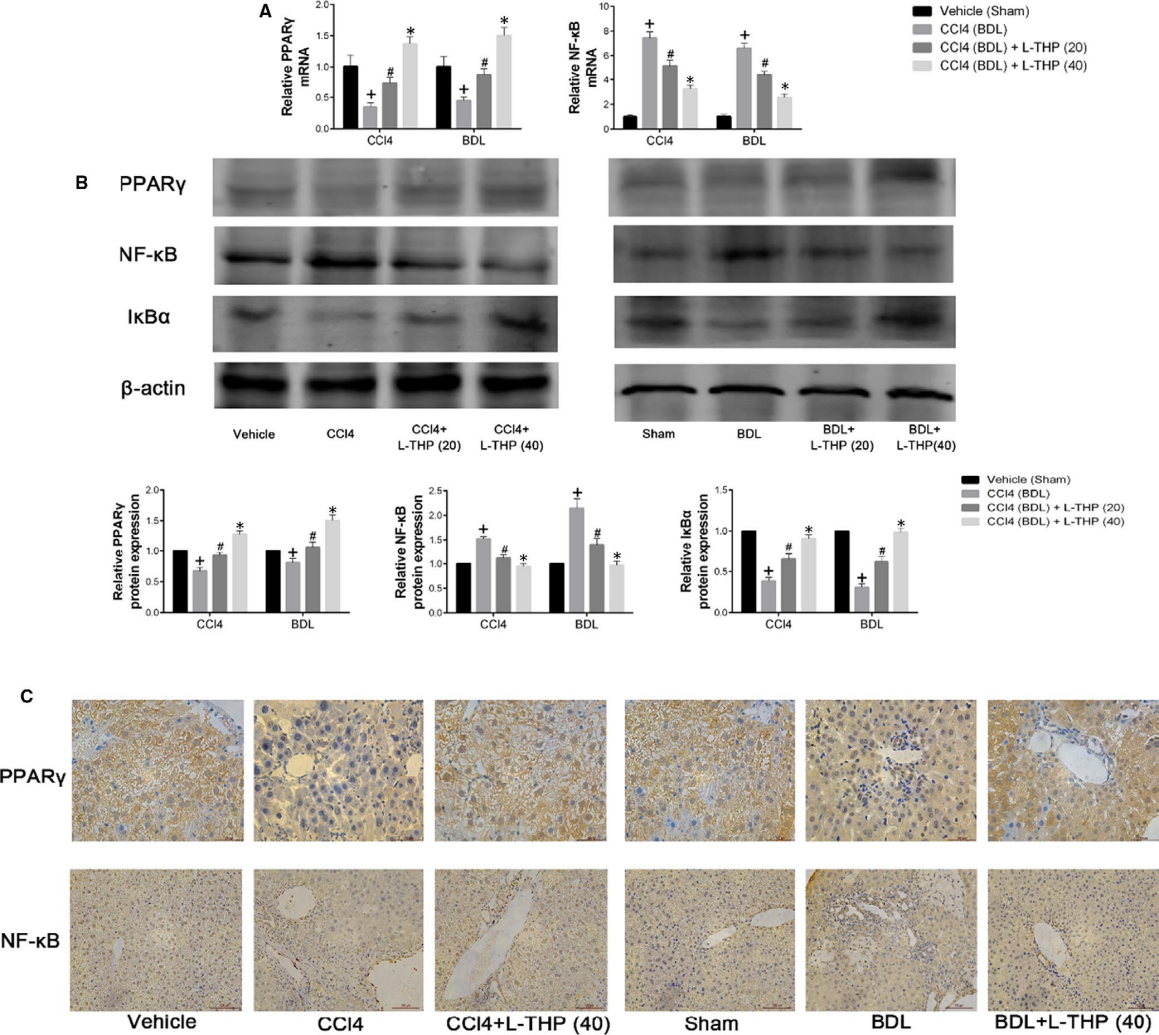
Effects of levo‐tetrahydropalmatine on the PPARγ/NF‐κB pathway in liver fibrosis: (A) The PCR analysis. L‐THP up‐regulated PPARγ while down‐regulated NF‐κB mRNA levels. Data are expressed as means ± SD (n = 3, ^+^ *P* < .05 for CCl4 or BDL vs Vehicle or Sham; *^#^P* for CCl4+L‐THP (20) or BDL+L‐THP (20) versus CCl4 or BDL; *P for CCl4 +L‐THP (40) or BDL+L‐THP (40) vs CCl4 +L‐THP (20) or BDL+L‐THP (20)). (B) Western blot and quantitative analysis of PPARγ, NF‐κB and IκBα. L‐THP increased PPARγ and IκBα protein expressions while reduced NF‐κB protein expressions in liver tissues. Data are expressed as means ± SD (n = 3, ^+^ *P* < .05 for CCl4 or BDL vs Vehicle or Sham; *^#^P* for CCl4+L‐THP (20) or BDL+L‐THP (20) vs CCl4 or BDL; *P for CCl4 +L‐THP (40) or BDL+L‐THP (40) vs CCl4 +L‐THP (20) or BDL+L‐THP (20)). C, Immunohistochemical staining indicated that 40 mg/kg L‐THP treatment increased PPARγ while inhibited NF‐κB expression in liver tissues (original magnification: ×400)

The release of TGF‐β1 and other inflammatory cytokines are mainly modulated by NF‐κB in the development of liver fibrosis. The activation of PPARγ in HSCs could block NF‐κB by inhibiting the translocation of NF‐κB to the nucleus.^33^ To explore the underlying mechanism by which L‐THP down‐regulated the TGF‐β1/Smad pathway, the effect of L‐THP on PPARγ/NF‐κB signalling was detected. As shown in Figure 6A and B, the mRNA and protein levels of PPARγ were reduced following CCl4 and BDL, which were reversed by L‐THP treatment in dose‐dependent manners. The increased mRNA and protein expression of NF‐κB (P65) were suppressed by L‐THP treatment dose‐dependently. The results of immunohistochemistry were in accordance with western blotting (Figure 6C). IκBα could inhibit the translocation of NF‐κB to the nucleus. The protein levels of IκBα were increased in CCl4 and BDL groups, while were inhibited by L‐THP in dose‐dependent manners (Figure 6B). These results demonstrated that L‐THP up‐regulated the PPARγ/NF‐κB pathway in liver fibrosis.

### 3.7 Levo‐tetrahydropalmatine suppressed the autophagy of HSCs and modulated PPARγ/NF‐κB and TGF‐β1/Smad pathway in vitro

**FIGURE 7:**
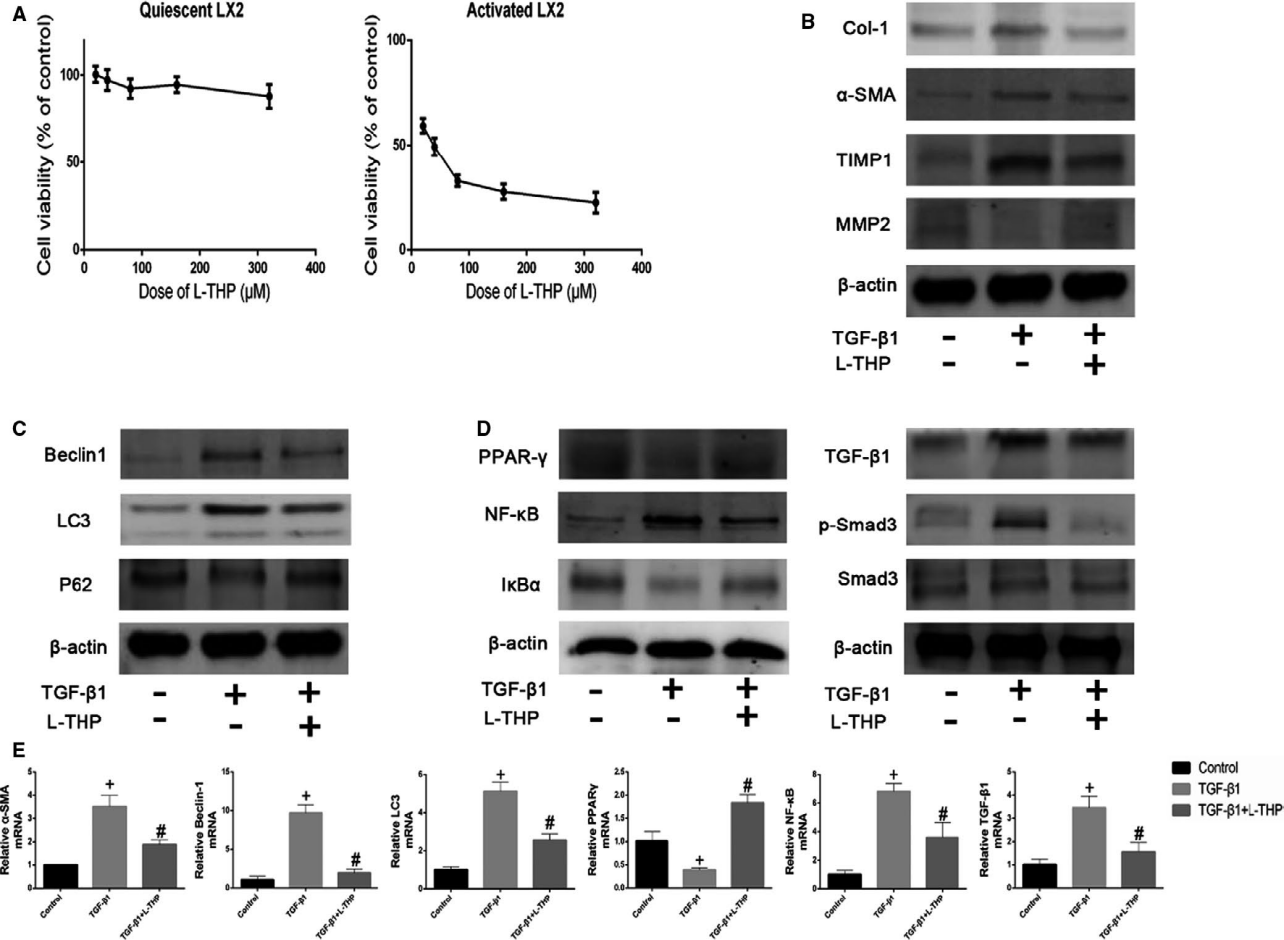
Effects of levo‐tetrahydropalmatine on the ECM synthesis, autophagy and PPARγ/NF‐κB and TGF‐β1/Smad pathway in LX2 cells in vitro: (A) Cell viability after L‐THP treatment in quiescent and activated LX2 cells (n = 3). B, C & D, The protein expressions of Col‐1, α‐SMA, TIMP1, MMP2, Beclin1, LC3, P62, PPARγ, NF‐κB, IκBα, TGF‐β1, Smad3 and p‐Smad3 were detected by Western Blotting. E, The mRNA expressions of α‐SMA, Beclin1, LC3, PPARγ, NF‐κB and TGF‐β1 were detected by PCR analysis. Data are expressed as means ± SD (n = 3, ^+^ *P* < .05 for TGF‐β1‐treated group versus Control group; *^#^P* for L‐THP‐treated group vs TGF‐β1‐treated group)

To further detect the anti‐fibrotic effect of L‐THP in vitro, an activated LX2 cell model was constructed. The results of the CCK8 assay indicated that L‐THP did not affect the proliferation of quiescent LX2 cells while significantly suppressed the proliferation of activated LX2 cells (Figure 7A). The half‐maximum inhibition concentration (IC50) of L‐THP on activated LX2 cells was 34.01 μmol/L, which was calculated with CalcuSyn software. L‐THP at the dose of 34 μmol/L was used in the subsequent experiments. The protein expressions of Col‐1, α‐SMA and TIMP1 were up‐regulated, while the protein expression of MMP2 was inhibited by TGF‐β1 treatment, which was reversed by L‐THP treatment (Figure 7B). In addition, the enhanced mRNA expression of α‐SMA after TGF‐β1 administration was inhibited by 34 μmol/L L‐THP (Figure 7E). These results showed that L‐THP suppressed the activation and ECM synthesis in activated LX2 cells in vitro. In addition, the mRNA and protein expressions of Beclin1 and LC3 were enhanced in activated LX2 cells, which were suppressed by L‐THP treatment (Figure 7C & E). And the suppressed protein expression of P62 was enhanced by L‐THP (Figure 7C). These results indicated that L‐THP could inhibit the autophagy of activated LX2 cells in vitro.

Then, Western Blot and PCR were employed to detect the effects of L‐THP on the PPARγ/NF‐κB and TGF‐β1/Smad pathway in vitro. The protein expressions of NF‐κB, TGF‐β1 and p‐Smad3 were up‐regulated, while the protein expressions of PPARγ and IκBα were down‐regulated in the TGF‐β1 treated LX2 cells. The changes of these markers on the protein level were reversed by the L‐THP treatment (Figure 7D). In addition, the mRNA expressions of NF‐κB and TGF‐β1 were suppressed while the mRNA expression of PPARγ was enhanced by L‐THP in the activated LX2 cells. Based on the results above, we can conclude that L‐THP could inhibit the activation, ECM synthesis and autophagy in activated HSCs by modulating PPARγ/NF‐κB and TGF‐β1/Smad signalling to exert its anti‐fibrotic effect in vitro.

## 4 DISCUSSION

Liver fibrosis, or liver scarring, is a liver compensatory response to chronic liver injuries induced by a variety of insults: viral infection, alcoholic liver disease, fatty liver diseases, drug abuse and autoimmune diseases. If uncontrolled, liver fibrosis may develop into liver cirrhosis and hepatocellular carcinoma.^34^ At present, the only curative strategy for liver fibrosis is liver transplantation, which is limited by the lack of donors. Novel and efficient drugs for liver fibrosis treatment are urgently needed.

Levo‐tetrahydropalmatine (L‐THP) is an active component of traditional Chinese medicine C. yanhusuo. L‐THP has been used as an analgesic and sedative drug for many years in China.^22^ In addition, L‐THP has been reported to have cardioprotective effect, antioxidative effect and antitumor effect.^24^, ^25^, ^26^ Our previous studies demonstrated that L‐THP suppressed hepatocyte apoptosis and autophagy to protect mice from liver injury induced by concanavalin A and hepatic ischaemia and reperfusion.^27^, ^28^ Moreover, Min et al. found that dl‐tetrahydropalmatine (dl‐THP) protected mice from acute liver injury induced by CCl4. The protective effect of dl‐THP was based on the inhibition of lipid peroxidation products.^31^ Yu et al. found that tetrahydropalmatine (THP) alleviated irradiation‐induced pulmonary fibrosis by inhibiting the collagen deposition in the lung.^35^ However, the effect of L‐THP on liver fibrosis remains undetermined.

To determine the anti‐fibrotic effect of L‐THP on liver fibrosis and underlying mechanisms, two animal liver fibrosis models (CCl4 and BDL) and a LX2 cell model were built in the present study. CCl4 and BDL animal models are considered stable models of human liver fibrosis.^7^ CCl4 can mimic human liver fibrosis induced by toxin or hepatitis B infection. BDL‐induced liver fibrosis is similar to human cholestatic‐induced periportal biliary fibrosis. In the present study, the results showed that 20 and 40 mg/kg L‐THP reduced serum ALT and AST levels and improved hepatic histological changes in both liver fibrosis models. 40 mg/kg L‐THP exerted a stronger hepatoprotective effect than 20 mg/kg L‐THP.

Liver fibrosis is characterized by the excessive deposition of ECM due to enhanced synthesis and insufficient degradation. In the liver, ECM is secreted by HSCs, hepatocytes, liver sinusoidal endothelial cells (SECs) and Kupffer cells. During liver fibrosis, activated HSCs are the main source of ECM.^36^ In physiological conditions, quiescent HSCs modulate the storage and metabolism of vitamin A. Following liver injury, HSCs are activated and transform into proliferative myofibroblasts (MFs), which is characterized by enhanced ECM synthesis, loss of lipid droplets and expression of unique molecular marker α‐SMA.^12^ α‐SMA is identified a marker of HSC activation. Collagen type I (Col‐1) is the principal component of ECM. Hydroxyproline is the characteristic amino acid of Col‐1, the hepatic levels of hydroxyproline indicate the severity of liver fibrosis. MMPs, a family of enzymes containing Ca^2+^ and Zn^2+^, modulate the degradation of ECM. TIMPs are specific inhibitors of MMPs and can promote the ECM synthesis.^37^ In physiological conditions, the balance between MMPs and TIMPs contributes to ECM homoeostasis in the liver, which is disturbed in liver fibrosis, leading to the excessive deposition of ECM.^8^ In the present study, L‐THP reduced the hepatic levels of hydroxyproline dose‐dependently in both fibrosis models. The results of PCR, western blotting and immunohistochemical staining indicated that L‐THP suppressed the mRNA and protein expressions of Col‐1, α‐SMA and TIMP1 while increased the expression of MMP2 in both fibrosis models. Moreover, results of Masson’s trichrome staining confirmed that L‐THP suppressed ECM deposition in the liver sections. Furthermore, L‐THP suppressed activation and ECM synthesis of activated LX2 cells in our in vitro study. These results demonstrated that L‐THP treatment inhibited HSCs activation, suppressed ECM synthesis and enhanced ECM degradation to exert its anti‐fibrotic effect.

Autophagy, or type II programmed cell death, is an intracellular metabolic process by which proteins, damaged organelles and microbes are degraded by lysosomes. Autophagy provided energy to promote cell survival in the presence of stress, hypoxia, inflammation and chemotherapeutic drugs. Autophagy is characterized by the formation of autophagosomes. Beclin1 is essential to the nucleation in the autophagy process. LC3 plays a significant role in the formation of autophagosomes. P62 is an autophagy adaptor that brings substrates to the autophagosomes and is degraded by autophagy.^38^ Beclin1, LC3 and P62 are identified as markers of autophagy. Autophagy has been reported to play a key role in the pathogenesis of a variety of liver diseases, including autoimmune hepatitis, liver IR injury, liver fibrosis and hepatocellular carcinoma.^3^, ^16^, ^39^, ^40^ Autophagy promotes the catabolism of lipid droplets into free fatty acids by stimulating β‐oxidation, producing adenosine triphosphate (ATP) to provide energy for HSCs activation^20^ and promote liver fibrogenesis. The inhibition of autophagy in HSCs by pharmacological inhibitors (3‐methyladenine or chloroquine) or knockdown of autophagy genes Atg7 or Atg5 blocked HSCs activation and ECM synthesis in vitro.^20^ The authors also reported that HSC‐specific deletion of Atg7 attenuated mice liver fibrosis induced by CCl4 or thioacetamide.^20^ Thoen et al found that an autophagy inhibitor bafilomycin A1 inhibited the activation and proliferation of human HSCs and mouse HSCs in vitro by blocking autophagy.^41^ These findings demonstrated the potential of autophagy as a target of liver fibrosis treatment. Our previous studies showed that L‐THP blocked autophagy to attenuate liver injury induced by concanavalin A and hepatic ischaemia and reperfusion.^27^, ^28^ In the present study, the mRNA and protein expressions of Beclin1, LC3 and P62 were detected by PCR, western blotting and immunohistochemical staining. The results showed that L‐THP suppressed HSCs autophagy dose‐dependently in both mouse liver fibrosis models. The results of TEM indicated that L‐THP suppressed the formation of autophagosomes in the liver sections from the CCl4 treated groups. In addition, the results of double‐immunofluorescence staining showed that L‐THP inhibited the expression of Beclin1 and LC3 in HSCs in the CCl4 treated mice livers. Furthermore, L‐THP inhibited autophagy in activated LX2 cells in vitro. These results indicated that the anti‐fibrotic effect of L‐THP was related to its inhibitory effect on HSCs autophagy.

The underlying mechanisms by which L‐THP inhibited the ECM synthesis and autophagy in liver fibrosis were further determined. In the presence of chronic liver injury, hepatocytes and Kupffer cells secreted various inflammatory cytokines, mainly TGF‐β1. TGF‐β1 binds to TGF‐β1 receptors expressed on HSCs to activate HSCs. Moreover, activated HSCs secret TGF‐β1, resulting in the persistent activation of HSCs.^42^ TGF‐β1 could bind to TGF‐β type Ⅱ receptor (TβRII) and then activate TGF‐β type I receptor (TβRI) through phosphorylation. Then, TGF‐β1 interacts with activated TβRI to phosphorylate downstream molecules Smad2 and Smad3. Phosphorylated Smad2 and Smad3 (p‐Smad2 and p‐Smad3) then translocate into and accumulate in the nucleus, modulating the transcription of target genes.^43^ TGF‐β1/Smad pathway plays a key role in the pathogenesis of liver fibrosis. It has been reported that activated TGF‐β1/Smad pathway promoted ECM deposition by enhancing TIMP1 expression and inhibiting MMP2 expression.^44^, ^45^ In addition, the TGF‐β1/Smad pathway has been reported to up‐regulate autophagy by modulating the transcription of Beclin1.^46^ In the present study, the results of PCR, western blotting and immunohistochemical staining showed that L‐THP reduced the expression of TGF‐β1 and the phosphorylation of Smad2/3 in both animal models and LX2 cell model. The inhibitory effects of L‐THP on HSCs activation, ECM deposition and autophagy were associated with the down‐regulation of the TGF‐β1/Smad pathway.

NF‐κB family members are identified as key regulators of innate and adaptive immune responses by modulating the transcription of target genes in the nucleus. In normal conditions, NF‐κB subunits bind to NF‐κB inhibitor IκBα to form IκBα/p50/p65 complex in the cytoplasm, preventing the translocation of NF‐κB into the nucleus. In the presence of stimuli, NF‐κB is activated and then translocated into the nucleus, leading to the transcription of target genes.^47^ It has been reported that NF‐κB takes part in the pathological mechanisms underlying liver fibrosis.^48^ In liver fibrogenesis, the transcription of TGF‐β1 and other inflammatory cytokines are mainly modulated by NF‐κB.^49^ Moreover, it has been reported that Kupffer cells (hepatic macrophages) promoted the survival of activated HSCs in a NF‐κB‐dependent manner, contributing to the development of liver fibrosis.^50^ In addition, Freudlsperger et al found that TGF‐β1 promoted the IκB kinase phosphorylation and degradation of IκBα, leading to the activation of NF‐κB.^51^ Feng et al reported that salidroside attenuated mice liver fibrosis induced by CCl4 and BDL by inhibiting the TGF‐β1/p‐Smad3 pathway. The inhibition of TGF‐β1/p‐Smad3 is based on the suppression of NF‐κB expression and nuclear translocation in Kupffer cells and HSCs in vivo.^1^ Shen et al found that in mice liver fibrosis models, astaxanthin decreased TGF‐β1 expression by inhibiting NF‐κB expression in the nucleus. Moreover, astaxanthin suppressed the expression of NF‐κB in macrophages in vitro.^2^ L‐THP was reported to suppress inflammatory responses by inhibiting the NF‐κB pathway.^28^, ^52^ In the present study, the results of PCR, western blotting and immunohistochemical staining indicated that L‐THP treatment decreased NF‐κB while enhanced IκBα expression in animal and LX2 cell models. Hence, it is reasonable to believe that L‐THP inhibited TGF‐β1 expression by down‐regulating NF‐κB pathway.

Peroxisome proliferator‐activated receptor‐γ (PPARγ) is a member of the nuclear receptor superfamily. PPARγ is mainly expressed in adipocytes, colon, and immune cells. PPARγ has been reported to exert a variety of biological functions, including modulation of lipid and carbohydrate metabolism, inflammatory response, cell proliferation and differentiation.^53^ Natural and synthetic PPARγ agonists have shown potential in the treatment of various liver diseases, including non‐alcoholic fatty liver disease (NAFLD), autoimmune hepatitis, liver fibrosis and hepatocellular carcinoma.^54^, ^55^ PPARγ is highly expressed in quiescent HSCs while its levels and trans‐activating activity are suppressed in activated HSCs.^56^ It has been reported that forced PPARγ expression in activated HSCs by adenoviral vector reversed activated HSCs to a quiescent phenotype at morphologic and biochemical levels, including retracted cytoplasm, prominent dendritic processes, reduced stress fibres and restored ability to accumulate retinyl esters. These protective effects could be inhibited by the expression of a dominant negative mutant of PPARγ. Forced PPARγ expression also reduced the expression of Col‐1, α‐SMA and TGF‐β1 in activated HSCs.^57^ Xu et al reported that curcumin suppressed proliferation of activated HSCs in vitro by inducing the expression of PPARγ. Curcumin treatment also leaded to the inhibition of NF‐κB trans‐activating activity and DNA binding activity. More importantly, the inhibitory effect of curcumin on NF‐κB trans‐activating activity could be reversed by a specific PPARγ antagonist PD‐68235 in a dose‐dependent manner.^33^ In a mice NAFLD model, L‐carnitine alleviated hepatic inflammation and fibrosis by inhibiting NF‐κB p65 in a PPARγ‐dependent manner.^58^ Hepatic macrophages, or Kupffer cells, are an important source of ECM, TGF‐β1 and other inflammatory cytokines in liver fibrosis.^59^ It has been reported that activated PPARγ suppressed macrophage activation and inflammatory response by inhibiting NF‐κB.^60^ Feng et al reported that apigenin activated PPARγ to reverse macrophage towards an anti‐inflammatory M2 phenotype. These protective effects were associated with PPARγ‐mediated inhibition of p65 translocation into nuclei.^61^ These findings demonsrated that PPARγ activation could inhibit the NF‐κB pathway and plays a significant role in hepatic fibrogenesis. In the present study, the results of PCR, western blotting and immunohistochemical staining showed that PPARγ expressions were hindered in CCl4 and BDL groups and activated LX2 cells, which were restored by L‐THP treatment. These results indicated that NF‐κB inhibition induced by L‐THP might be based on the activation of PPARγ.

**FIGURE 8:**
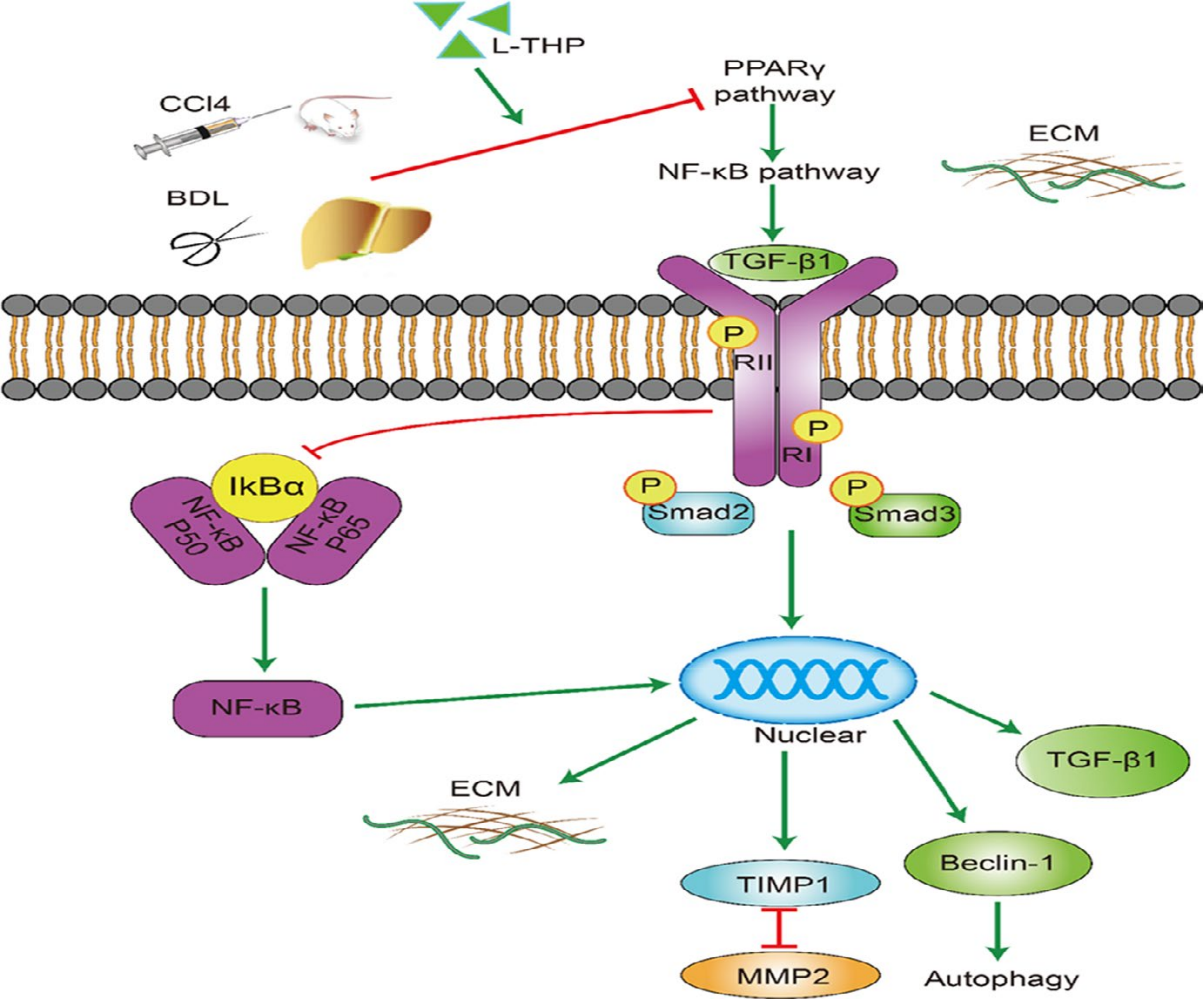
Protective mechanism of levo‐tetrahydropalmatine against liver fibrosis. L‐THP up‐regulated PPARγ expression, leading to the inhibition of NF‐κB pathway. Inhibited NF‐κB down‐regulated the expression of TGF‐β1 and TGF‐β1/Smad pathway, suppressing HSCs activation, ECM synthesis and autophagy

The present study collectively demonstrates that L‐THP exerts an anti‐fibrotic effect in CCl4 and BDL mouse models and activated the LX2 cell model. L‐THP modulates the PPARγ/NF‐κB pathway to inhibit TGF‐β1 expression in vivo and in vitro. Inhibition of TGF‐β1 down‐regulated downstream TGF‐β1/Smad pathway, resulting in the inactivation of HSCs, reduced ECM deposition and inhibition of autophagy (Figure 8). The safety of L‐THP for clinical application and the effect of L‐THP on hepatocellular carcinoma should be further investigated.

## 5 CONCLUSION

The present study confirmed the anti‐fibrotic effect of L‐THP in CCl4 and BDL mice liver fibrosis models and activated LX2 cell model. L‐THP reduced TGF‐β1 expression in the liver by regulating the PPARγ/NF‐κB pathway. PPARγ activation by L‐THP inhibited the NF‐κB pathway, suppressing HSCs activation, ECM synthesis and autophagy by down‐regulating the downstream TGF‐β1/Smad pathway.

## CONFLICT OF INTERESTS

The authors declare no conflicts of interest.

## AUTHOR CONTRIBUTIONS

Qiang Yu and Ping Cheng designed the research. Qiang Yu performed the animal experiments. Ping Cheng performed data analysis. Chuanyong Guo and Jianye Wu edited the manuscript.

## Supporting information

Figure S1

Table S1

## Data Availability

The datasets generated during and/or analysed during the current study are available from the corresponding author on reasonable request.
