# Supplementary material for: PPARγ/NF‐κB and TGF‐β1/Smad pathway are involved in the anti‐fibrotic effects of levo‐tetrahydropalmatine on liver fibrosis
Source: J Cell Mol Med. 2021 Jan 12;25(3):1645–60. doi: 10.1111/jcmm.16267 (PMC7875896; doi:10.1111/jcmm.16267)
Supplement: Supplementary file 1 — Figure S1 [file JCMM-25-1645-s001.pdf]

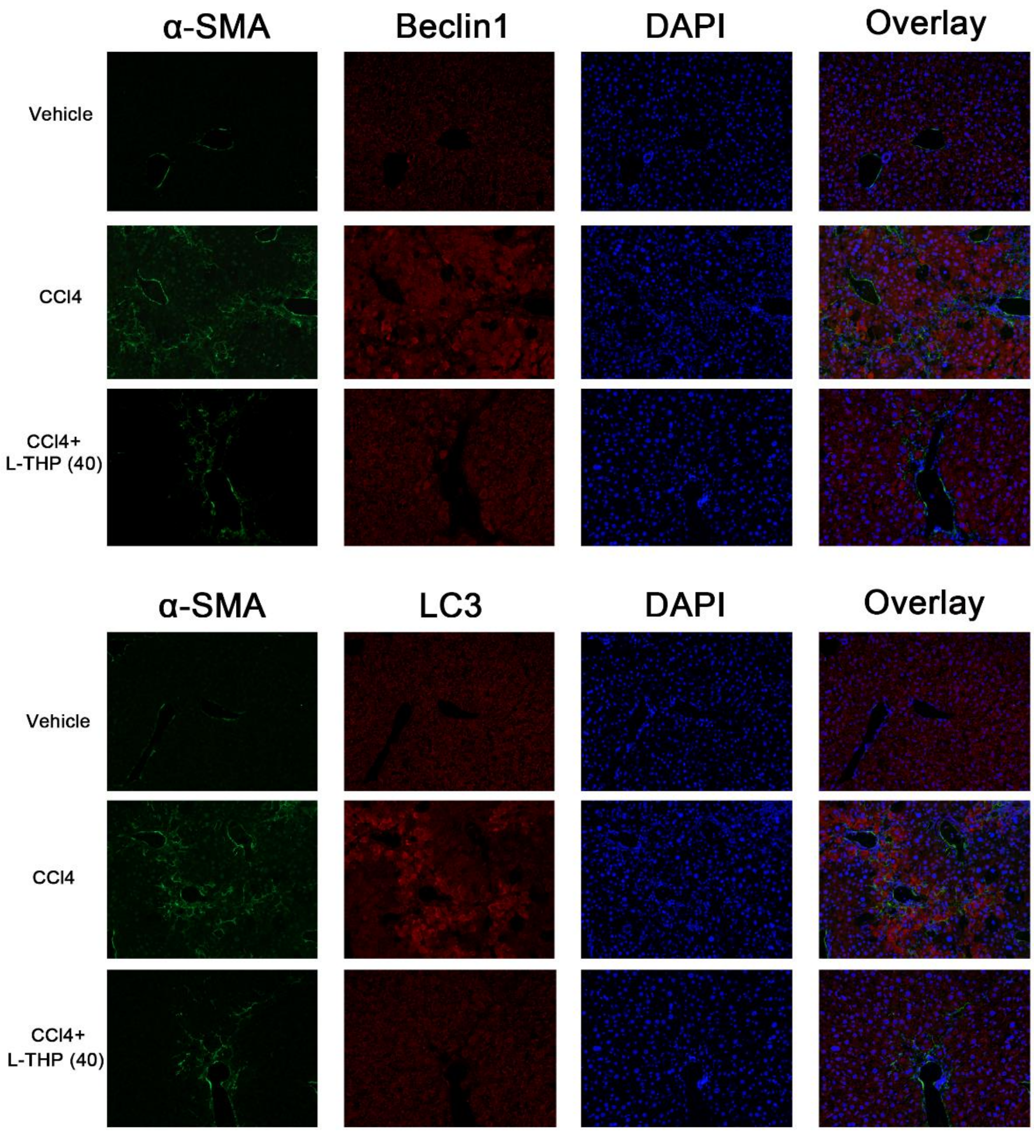

Figure S1:Representative images of double-immunofluorescence staining of liver sections. α-SMA is considered a marker of activated HSCs. 40 mg/kg L-THP suppressed the protein expressions of Beclin1 and LC3 in activated HSCs (original magnification: ×400) .
