# Supplementary material for: PPARγ/NF‐κB and TGF‐β1/Smad pathway are involved in the anti‐fibrotic effects of levo‐tetrahydropalmatine on liver fibrosis
Source: J Cell Mol Med. 2021 Jan 12;25(3):1645–60. doi: 10.1111/jcmm.16267 (PMC7875896; doi:10.1111/jcmm.16267)
Supplement: Supplementary file 2 — Table S1 [file JCMM-25-1645-s002.pdf]

Supplementary Table 1: Sequences of primers (Human) used for RT-PCR

Gene	DNA strand	Primer sequence (5′-3′)
β-actin	Forward<br>Reverse	CATGTACGTTGCTATCCAGGC<br>CTCCTTAATGTCACGCACGAT
α-SMA	Forward<br>Reverse	AAAAGACAGCTACGTGGGTGA<br>GCCATGTTCTATCGGGTACTTC
Beclin-1	Forward<br>Reverse	ACCTCAGCCGAAGACTGAAG<br>AACAGCGTTTGTAGTTCTGACA
LC3	Forward<br>Reverse	AAGGCGCTTACAGCTCAATG<br>CTGGGAGGCATAGACCATGT
NF-κB	Forward<br>Reverse	GTGGGGACTACGACCTGAATG<br>GGGGCACGATTGTCAAAGATG
PPARγ	Forward<br>Reverse	ACCAAAGTGCAATCAAAGTGGA<br>ATGAGGGAGTTGGAAGGCTCT
TGF-β1	Forward<br>Reverse	GGCCAGATCCTGTCCAAGC<br>GTGGGTTTCCACCATTAGCAC
